# *Cis* regulatory motifs and antisense transcriptional control in the apicomplexan *Theileria parva*

**DOI:** 10.1186/s12864-016-2444-5

**Published:** 2016-02-20

**Authors:** Kyle Tretina, Roger Pelle, Joana C. Silva

**Affiliations:** Institute for Genome Sciences and Department of Microbiology and Immunology, University of Maryland School of Medicine, Baltimore, MD USA; International Livestock Research Institute (ILRI), Nairobi, Kenya

**Keywords:** *Theileria parva*, Transcription regulation, Regulatory motifs

## Abstract

**Background:**

*Theileria parva* is an intracellular parasite that causes a lymphoproliferative disease in cattle. It does so by inducing cancer-like phenotypes in the host cells it infects, although the molecular and regulatory mechanisms involved remain poorly understood. RNAseq data, and the resulting updated genome annotation now available for this parasite, offer an unprecedented opportunity to characterize the genomic features associated with gene regulation in this species. Our previous analyses revealed a *T. parva* genome even more gene-dense than previously thought, with many adjacent loci overlapping each other, not only at the level of untranslated sequences (UTRs) but even in coding sequences.

**Results:**

Despite this compactness, *Theileria* intergenic regions show a pattern of size distribution indicative of monocistronic gene transcription. Three previously described motifs are conserved among *Theileria* species and highly prevalent in promoter regions near or at the transcription start sites. We found novel motifs at many transcription termination sites, as well as upstream of parasite genes thought to be critical for host transformation. Adjacent genes that could be regulated by antisense transcription from an overlapping transcriptional unit are syntenic between *T. parva* and *P. falciparum* at a frequency higher than expected by chance, suggesting the presence of common, and evolutionary old, regulatory mechanisms in the phylum Apicomplexa.

**Conclusions:**

We propose a model of transcription with conserved sense and antisense transcription from a few taxonomically ubiquitous and several species-specific promoter motifs. Interestingly, the gene networks regulated by conserved promoters are themselves, in most cases, not conserved between species or genera.

**Electronic supplementary material:**

The online version of this article (doi:10.1186/s12864-016-2444-5) contains supplementary material, which is available to authorized users.

## Background

*Theileria parva* infection induces a lymphoproliferative disease, called East coast fever (ECF), in cattle in eastern, central, and southern Africa, where it causes > $300 M USD of economic loss per year [1]. *T. parva* sporozoites in the salivary glands of ticks are introduced into the bloodstream of cattle, where they infect lymphocytes. In the lymphocyte cytoplasm, sporozoites develop into multinucleate schizonts, which transform the infected cell to exhibit several cancer-like phenotypes, including hyper-proliferation and replicative immortality [2]. The parasite genes regulating these processes are only beginning to be uncovered. However, our understanding of how those genes are regulated remains limited by the lack of knowledge regarding transcriptional regulation in *T. parva*. No genetic modification systems have been developed for *T. parva*, and only recently has a successful transfection system been reported [3], making it exceedingly difficult to investigate gene function and regulation by standard molecular biology approaches in this parasite. Bioinformatics methods provide a powerful complementary approach to molecular biology in the characterization of mechanisms of gene regulation [4].

The identification and characterization of core promoters and transcription start sites is key to understanding gene regulation in any organism [5]. Although early models of RNA polymerase II transcription suggested that a core promoter, such as a TATA box, directs transcription from a single defined nucleotide position, recent genome-wide studies favor a more intricate model that includes multiple promoters and start sites for most genes. This is a potentially significant source of transcriptome diversity [6]. Apicomplexans are notable for their lack of canonical eukaryotic transcription factors, and until the relatively recent discovery of the ApiAP2 transcription factor family it was thought that transcription was not a very important mechanism of gene regulation in apicomplexans [7–9]. While *Plasmodium* species now have extensive transcription factor binding site information for the ApiAP2 family [10], no such resource exists for *T. parva*, even though it could significantly enrich further research and an understanding of its pathogenesis. Interestingly, in *Plasmodium*, the DNA binding motifs for orthologous ApiAP2 transcription factors are conserved across the genus, but the genes that they regulate are not [10]. It is not, however, known if this is the case within Piroplasmida, the order to which *Theileria* and *Babesia* species belong. Here, we use the first published RNAseq dataset for *T. parva*, as well as a variety of bioinformatics methods to predict *cis* regulatory motifs in *T. parva* and other piroplasmids, and investigate the sequence and evolution of genomic motifs that regulate gene expression.

## Results and discussion

Aside from early bioinformatics predictions of potentially functional *cis* regulatory motifs in the *T. parva* genome [11], very little is known about the basic transcriptional unit in this species. Those analyses revealed that in *T. parva* 5′5′ intergenic regions (i.e., those flanked by genes in head-to-head orientation) are significantly longer than 5′3′ intergenic regions (genes head-to-tail), which in turn are significantly longer than 3′3′ intergenic regions (genes tail-to-tail), a clear signature of spatial requirements imposed by upstream regulatory motifs in a very compact genome [11–13]. A similar pattern is also found in fungal genomes [14, 15]. Those analyses were based only on a small subset of genes in the original annotation of the *T. parva* genome [11, 16], and without knowledge of the location of transcription start sites. The extensive *T. parva* RNAseq data recently made available in Genbank formed the basis for the improved re-annotation of its genome (Tretina and Silva, in prep) and provides information on the start and end sites of transcripts and UTR composition in this species. The updated genome annotation, with close to 50 % of the genes with revised coding sequences, together with the RNAseq data form, a powerful dataset to mine for core promoters and other gene transcription regulatory motifs. Even though the motifs identified here are based solely on *in silico* analyses, we attempt to provide potential biological context and extrapolate on potential implications by drawing parallels with similar observations in other taxa.

### Potentially functional *cis* regulatory motifs include G-box and Spe2 and motifs near transcription termination sites of genes expressed in the *T. parva* schizont stage

**Fig. 1:**
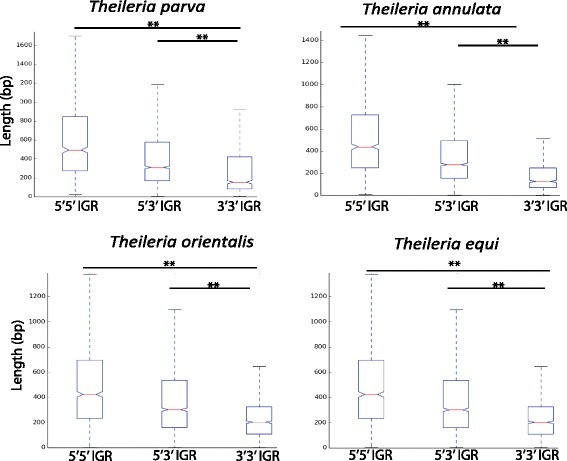
Length of the three types of intergenic region (IGR) length in the genomes of *T. parva, T. annulata, T. orientalis,* and *T. equi*. IGR length, shown in base pairs, correlates with the directionality of the transcriptional units that flank them. Boxplot shows median (orange line), second and third quartiles (within box) and max value below 75 % + 1.5 interquartile range and minimum value above 25 % - 1.5 interquartile range (whiskers). Comparisons of intergenic region types within each species were significantly different than each other (0.001 < *p* < 0.005, two-tailed Student’s *t*-test)

We now expand the original *T. parva* analyses to the whole genome, and investigated similar patterns in the other three *Theileria* species for which a genome assembly is publicly available, namely *T. annulata*, *T. orientalis* and *T. equi* [16–19]. As would be expected for a genome that includes primarily monocistronic transcriptional units, the ratio of 5′5′ : 5′3′ : 3′3′ intergenic regions is approximately 1:2:1, respectively (Table 1). The rather small size of the intergenic regions, and the correlation between their length and the number of flanking 5′ gene ends, indicate that genome space is tightly regulated and at a premium in these organisms, with longer intergenic regions reflecting increased functionality added by *cis*-acting elements (Table 1, Fig. 1).

**Table 1:** Comparisons of the number, type, and size of intergenic regions (IGR) in four sequenced *Theileria* parasites (*T. parva*, *T. annulata*, *T.orientalis,* and *T. equi*)

Species	IGR Type^b^	*n*	Ratio of IGR types^b^	Median Size	Ratio of Median Size^b^
*T. parva*	5'5'	851	1.00	493	3.21
	5'3'	1968	2.30	309	2.01
	3'3'	854	1.00	154	1.00
*T. annulata*	5'5'	908	1.00	438	3.49
	5'3'	1949	2.14	277	2.21
	3'3'	910	1.00	126	1.00
*T. orientalis*	5'5'	1056	1.00	424	2.09
	5'3'	1838	1.74	302	1.49
	3'3'	1054	1.00	203	1.00
*T. equi*	5'5'	1203	1.00	645	4.76
	5'3'	2853	2.38	394	2.91
	3'3'	1198	1.00	136	1.00

^a^IRG type: genes in head-to-head (5'5'), head-to-tail (5'3') and tail-to-tail (3'3') orientation. ^b^Ratio used as a reference (denominator) the value for 3'3' IGRs

We then searched for additional *cis*-regulatory motifs in the *T. parva* genome. The extremely biased content in intergenic regions in *Plasmodium*, with GC nucleotide content close to 10 %, hinders the application of some motif-finding tools [20]. *Theileria* species do not have the same limitation, since they have considerably smaller intergenic regions, with higher GC content. *De novo* motif searches in *Toxoplasma gondii*, which has a relatively GC-rich genome, identified potential *cis* regulatory motifs that are different from any that have been identified in *T. parva* [21]. Here, specific genomic regions were extracted and motifs found to be enriched in these sequences were identified *de novo* using the MEME suite. The distribution of these motifs relative to transcription or translation initiation or termination sites was determined with FIMO, and their distribution in different genomic regions was determined with MAST (Methods).

#### Upstream motifs

**Fig. 2:**
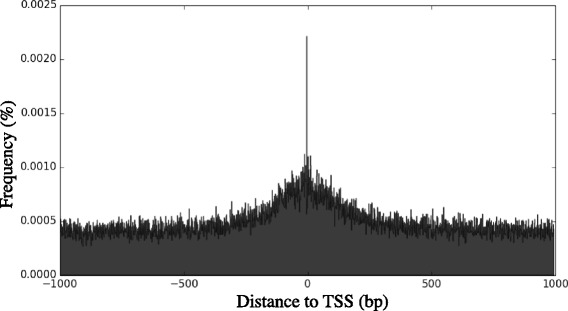
Distribution of the TYAYWWW motif around transcription initiation sites. The horizontal axis represents sequence areas from -1,000 bp to +1,000 bp around transcription initiation sites with single nucleotide resolution. Position 0 represents the transcription initiation site, with a peak observed at the -3 position. The vertical axis represents the normalized frequency of the motif

Recent work in the piroplasma species *Babesia bovis* has identified the consensus sequence TYAYWWW with a tight distribution around transcription start sites, that suggests similarity to an initiator-like motif [22]. As validation to our updated gene models, we have also found this motif with start site peaking in distribution 3 bp upstream of transcription start sites in the *T. parva* genome (Fig. 2).

**Fig. 3:**
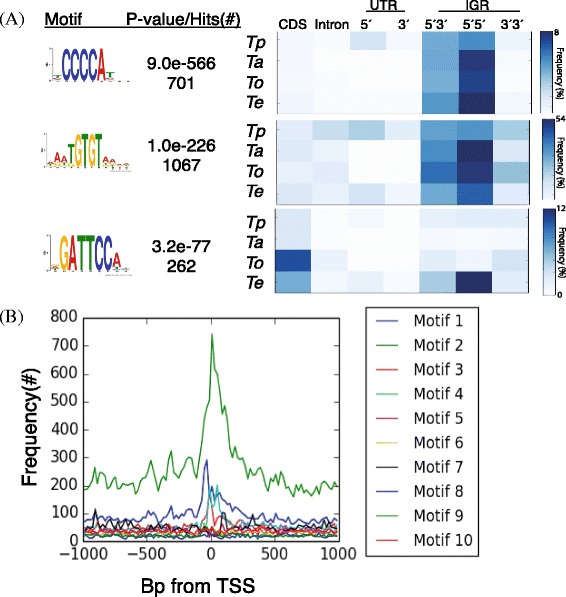
Metrics of the top 3 motifs identified by MEME searches around transcription initiation sites. **a** The top three motifs (G-box, Spe2, and NFkB-like), their p-value identified by MEME, and their frequency in different genomic regions of four *Theileria* species and in the three types of intergenic regions (IGR) (*Tp* = *Theileria parva*; *Ta* = *Theileria annulata*; *To* = *Theileria orientalis; Te* = *Theileria equi*). **b** Distribution of the three motifs in the vicinity of transcription initiation sites in *T. parva*. All three motifs are located predominantly at or near transcription start sites (TSS)

Since the typical size of promoter regions in *T. parva* is unknown, MEME searches were conducted on genomic sequences of 50, 100, and 150 base pairs centered on the annotated transcription start site of all mRNAs that do not overlap other transcripts. Many identical motifs were identified in all three searches, with three distinct motifs found among the top four (Additional file 1: Figure S1), suggesting a tight distribution around the transcription initiation sites. Three of these motifs were previously identified [11], and our expanded analyses find additional information on their localization: in particular, two of the motifs were found predominantly near or at annotated transcript start sites, and were located most often in 5′5′ intergenic regions compared to other intergenic regions, coding regions, UTRs or introns, a pattern that holds true in all four *Theileria* species with an annotated genome assembly (Fig. 3, Additional file 2: Table S1).

One of these motifs, CCCCAT, shares a high level of similarity to the G-box motif found to be functional upstream of heat-shock proteins in *Plasmodium* species. This motif is bound by the *P. falciparum* ApiAP2 transcription factor PF13_0235_D1 [10], which does not have a bioinformatically identifiable homolog in *T. parva*. PF13_0235_D1 was found to be upstream of ribosomal and proteins involved in the unfolded protein response, indicating that this transcription factor regulates a biologically critical gene network. This motif has a very localized distribution in *B. bovis*, where it peaks 50 bp upstream of transcriptional start sites and has been implicated as an essential part of the core promoter [22]. A protein in *C. parvum* (cgd8_810) binds this motif, but the domain ortholog in *T annulata* (TA12015) does not [23]. A divergence in the function of the gene network regulated by the G-box motif in *T. annulata* is also indicated by the finding that it is enriched in promoters of genes that are up-regulated from the merozoite to piroplasm stages [23].

The second motif, AATGTGTAA, consists of a completely conserved core of five thymine and guanine nucleotides, with the two flanking and more labile adenines at each end. The core is identical to a motif over-represented in *B. bovis* promoter regions [22]. Interestingly, the orthologs TA11145 of *T. annulata* and PF3D7_0802100 (formerly MAL8P1.153) of *P. falciparum*, both have been shown to bind this motif [23]. In fact, a family of transcription factors conserved throughout the haematosporidians have been predicted to be auto-regulated and compete for binding to these motifs as a critical step in the stochastic differentiation process from macroschizont to merozoite life cycle stages [23]. A third motif, GATTCCA, is similar to the motif bound by NFkB in mammals [24]. Previous work has established this motif as being enriched upstream of genes involved in protein fate (i.e. post-translational modification, degradation/stabilization, and targeting), and a BLASTP analysis identified a *T. parva* protein, TP02_0125, to have and domain with 55 % identity to the DNA-binding domain of NFkB and therefore predicted to bind this motif [11]. Our BLASTP searches also identify strong homology (>80 % identity over >70 % of the gene) to proteins in *T. annulata* (TA11490), *T. orientalis* (TOT_020000113), and *B. bovis* (BBOV_IIII010230), but not in *T. equi,* where the best hit (BEWA_051930) had only 41 % identity over 13 % of the gene. Interestingly, and possibly not coincidentally, this third motif was not found in *T. equi*.

#### Downstream motifs

**Fig. 4:**
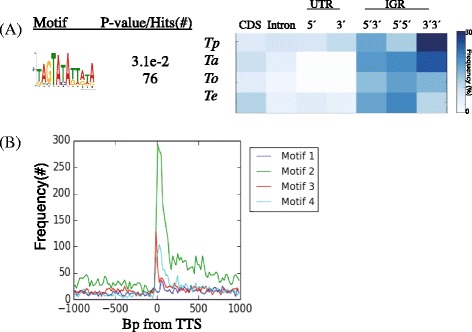
Metrics of the top motif identified by MEME searches of transcription termination sites. **a** Motif 2 was conserved in tail-to-tail intergenic regions in four *Theileria* species (*Tp* = *Theileria parva*; *Ta* = *Theileria annulata*; *To* = *Theileria orientalis; Te* = *Theileria equi*) and **b** peaked in distribution at transcription termination sites genome-wide in *T. parva*. The horizontal axis represents sequence areas from -1,000 bp upstream to +1,000 bp downstream around transcription initiation sites with single nucleotide resolution. Position 0 represents the transcription termination site. The vertical axis represents the frequency of the motif

The region downstream of genes may contain motifs involved in both termination of sense transcripts and the initiation of antisense transcripts encoded in the opposite strand. Antisense transcripts can arise from promoters near the terminal ends of their sense counterparts, and their expression is regulated by many of the same factors as sense genes [25]. Eukaryotic transcription termination is typically assumed to use the same mechanisms as model yeast systems, where the RNA polymerase II transcribes well past the polyadenylation signal (PAS), but a series of allosteric protein-protein interactions result in the cleavage of the transcript 11-30 bp downstream of the PAS and the degradation of the remaining RNA by endonucleases [26, 27]. Since there are no *cis* regulatory motifs reported to be involved in antisense transcription or transcription termination in *T. parva,* we searched for motifs around the annotated 3′ ends of transcripts. To this end, MEME searches similar to those described for promoter regions were conducted around transcript termination sites of annotated mRNAs. A few motifs were found to be over-represented around the 3′ end of transcripts, some of which were AT-rich and/or conducive to forming hairpin-like structures (Additional file 1: Figure S2c). One of these motifs (Additional file 1: Figure S2a, 50 bp region, motif 4) was identified in all 3′ motif searches, was enriched in 3′3′ intergenic regions of all four sequenced *Theileria* species (Fig. 4a, Additional file 2: Table S1), and was absent around transcription initiation sites. This AT-rich motif had pronounced peaks in distribution at transcript 3′ ends (Fig. 4b). Given the high AT-content and self-complementary nature of these motifs, with their hairpin potential, it could also be postulated that they may play a structural role in transcription termination. Interestingly, another of the motifs (Additional file 1: Figure S2a, 25 and 50 bp regions, motif 1) is identical to the central region of the second upstream *cis*-regulatory motifs described above. Therefore, it is tempting to postulate a possible double role for this motif in both transcription termination and antisense transcription initiation.

3′ UTRs also can play an additional significant role in gene regulation [28, 29] by potentially containing a signal for polyadenylation. Since the RNAseq protocol used in the present study included the enrichment of polyadenylated transcripts, we then took advantage of this dataset in order to predict possible canonical and non-canonical polyadenylation signals (PAS). It should be noted that non-polyadenylated, high-AT content parasite transcripts might also be pulled down using this protocol. With a previously established algorithm [30], we discovered that *T. parva* most likely uses a PAS that closely resembles, but is not identical to, that of other eukaryotes, including mammals, and found several other hexamers that we predict to play a role in polyadenylation (Additional file 2: Table S2).

#### UTR motifs and non-coding exons

Motifs found in 5′ UTRs contribute to post-transcriptional gene regulation in a number of organisms [31]. In *T. parva,* annotated 5′ UTRs, with median size of close to 150 bp, are significantly longer than 3′ UTRs, of median length <50 bp (Additional file 1: Figure S3), a pattern also found in *Plasmodium* and *Babesia* [13, 22]. Together with the presence of >100 RNA binding proteins in *T. parva* (Additional file 2: Table S3), this pattern is highly suggestive of a functional role for 5′ UTRs. In order to identify potential regulatory motifs encoded in UTRs, four separate motif searches were conducted: searches in 5′ and 3′ UTRs longer than 8 bp, each separated into those that either did or did not overlap an adjacent gene model. Out of these four UTR sets, conserved motifs were only identified in 5′ UTRs that do not overlap other transcripts (Additional file 1: Figure S4a, all motifs summarized in Additional file 1: Figure S4b). The motifs identified in these “non-overlapping” 5′ UTRs are considerably longer than the potential regulatory motifs found near transcription start sites and are rich in G and C nucleotides (GC-rich). The GC content of UTRs can affect protein translation efficiency; elevated GC content is associated with a decrease in translation efficiency [32]. The fact that the motifs found in these regions are GC-rich may indicate their role in post-transcriptional regulation.

**Fig. 5:**
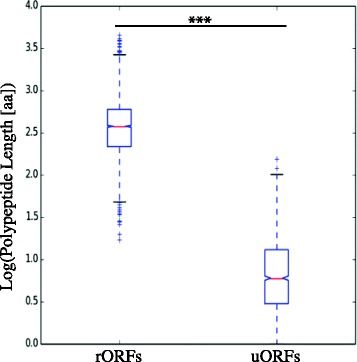
Length distribution of upstream open reading frames (uORFs) and reference open reading frames (rORFs). Only ORFs starting with a methionine were included in these distributions. Legend as in Fig. 1, with outliers are represented by ‘+’. Boxplot shows median (orange line), second and third quartiles (within box) and max value below 75 % + 1.5 interquartile range and minimum value above 25 % - 1.5 interquartile range (whiskers). uORF lengths were significantly lower than than rORF lengths (*p* < 0.001, two-tailed Student’s *t*-test)

Another mechanism by which 5′ UTRs have been found to impact gene expression is through the synthesis of small, upstream open reading frames (uORFs). In *Arabidopsis,* uORFs are present upstream of genes that encode regulatory products such as transcription factors [33]. Recent evidence suggests that they might play a role in translation delay in *P. falciparum*, perhaps by delaying translation of the main ORF or by encoding regulatory peptides themselves [34]*.* Small peptides translated from uORFs could also possibly be presented antigens on host MHC I, just like host defective ribosomal products [35], making uORFs important features to report. At least 5 % of *T. parva* genes have potential uORFs; 217 genes have at least one uORF that starts with a methionine, often considered the minimal ORF length for *bona fide* protein-coding gene, indicating that these peptides might play a significant role in *T. parva* biology (Fig. 5).

### Antisense transcription is highly prevalent in the *T. parva* genome and constitutes a potentially conserved mechanism of gene regulation

Antisense transcripts can play a role in the *cis* regulation of sense gene expression via epigenetic, transcriptional, post-transcriptional, or even translational interference mechanisms. They can also play *trans* regulatory roles in the expression of other genes in the genome [25]. Overlapping transcripts encoded on opposite strands, which are widespread in the *T. parva* genome and can result from run-through transcription (Tretina and Silva, in prep), have the potential to affect the expression level of neighboring gene when RNA polymerases transcribing in opposite directions collide [36, 37]. It is important to note that the temporal expression of overlapping genes cannot be determined from the RNAseq data used in the present study, since the RNA was extracted from unsynchronized cells.

Given the large percentage of overlapping transcripts from opposite strands in this genome (Tretina and Silva, in prep), the question arises of whether overlapping antisense transcription is merely “leakage” of the transcription machinery in a highly compact genome, or if instead it is the reflection of a carefully orchestrated transcriptional process, molded by selective pressure to optimize the level and timing of gene expression. Previous studies in humans have taken a comparative genomics approach to this question, assuming that if consecutive pairs of genes encoded on opposite strands improve organismal fitness (e.g., by temporal expression interference) then there will be strong selective pressure to preserve their synteny. In fact, they found that 23.3 % of human adjacent gene pairs encoded in opposite strands (i.e., those in tail-to-tail or head-to-head orientation), with overlapping transcripts, have conserved synteny in the pufferfish *Takifugu rubripes*, compared to only 13.5 % of consecutive head-to-tail gene pairs, i.e., those encoded in the same DNA strand [38]. We find similar evidence in *T. parva*: while of the 805 head-to-tail consecutive gene pairs in *T. parva* Muguga with orthologs in *P. falciparum* only 16 % have the same syntenic position in the genome of the latter species, the proportion is larger for pairs of consecutive genes encoded in opposite strands (23 %) and more than doubles to 35 % for genes pairs with evidence of potential transcriptional interference (Table 2). Therefore, we hypothesize that antisense overlapping of transcripts constitutes an important mechanism of transcriptional regulation in *T. parva*. Antisense transcripts are increasingly recognized as a vital part of gene regulation and antigenic variation in *Plasmodium* [39], particularly in the regulation of expression of virulence genes [40]. Given the paucity of identifiable transcription factors in these parasites [7, 41], antisense transcription may play a much more prominent role in gene regulation in apicomplexans than previously appreciated.

**Table 2:** Conservation of orientation of homologous gene pairs between *Theileria parva* Muguga and *Plasmodium falciparum* 3D7

Classification of *Theileria* gene pairs^a^	*Theileria* consecutive gene pairs with orthologs in *P. falciparum* ^c^	Pairs in *Theileria* with conserved synteny in *Plasmodium* ^b^	Percent conserved pairs^d^
Opposite strand	940	64	22.66
Opposite strand Overlap	182	64	35.16
Same-strand	805	125	15.53

^a^Classification of groups of *Theileria* consecutive gene pairs. Opposite strands: pairs of genes in head-to-head or tail-to-tail orientation, according to the 2014 *T. parva* Muguga annotation. Antisense: gene pairs for which a sesne-antisense relationship was observed in RNAseq data. Same strand: pairs of consecutive genes that are encoded on the same strand (head-to-tail orientation). ^b^Pairs of consecutive orthologous genes that preserved their gene order and orientation in *T. parva* Muguga and *P. falciparum* 3D7. ^c^Pairs of genes that are consecutive on the *T. parva* Muguga genome and have orthologs in the *P. falciparum* 3D7 genome. ^d^ Percentage of linked pairs

### Unique *cis* regulatory motifs regulate multi-gene families that are potentially involved in host pathogenesis and parasite immune evasion

Many apicomplexans have lineage-specific, telomeric multigene families which are often involved in host-pathogen interactions and evasion of the host immune system [42]. In *T. parva*, the Subtelomere-encoded Variable Secreted Protein (SVSP) family is the largest gene family and has been suggested to play a role in either immune evasion or the manipulation of host cell gene expression [2, 16, 43]. Another gene family, the *T. parva* Host Nucleus (TpHN) family, is homolous to a family of genes in *T. annulata* which have been shown to localize to the host nucleus and alter host gene expression [17, 44]. Given the interest in these gene families as potentially significant in the pathogenesis of ECF, we ran motif searches upstream of all genes in each of the top 20 gene families. Since little is known about the regulation of their expression, predictions of *cis* regulatory motifs that control the expression of these genes can provide rich information for future investigations. MEME searches of potential promoter regions of these genes revealed novel motifs that vary significantly from the motifs found in the previously mentioned motif searches (Additional file 2: Tables S4-S19). For the SVSP and TpHN gene families, several of these motifs showed conserved distribution relative to transcription initiation sites (Additional file 2: Table S20) as well as conserved enrichment in 5′5′ intergenic regions (Additional file 2: Tables S21-S25), indicating that they are likely functional and specific to the regulation of these genes, possibly acting in concert with the most common *T. parva* upstream motifs mentioned above, which are also found associated with many genes in these families.

### Gene regulatory motifs and their corresponding networks are not well conserved within piroplasmids

A set of regulatory motifs and the genes they regulate can be maintained through evolutionary time, or they may evolve in one of several ways: 1) a motif sequence may remain conserved, but the genes it regulates vary through time; 2) the motif sequence may evolve while the genes it regulates remain constant; 3) there is both a change in (or deletion of) the motif sequence and a rewiring of the networks of co-regulated genes. DNA binding motifs for orthologous ApiAP2 transcription factors are conserved between *P. falciparum* and *Plasmodium vivax*, although the genes that they regulate are not [10], reminiscent of scenario (1) above. This conservation occurs despite the fact that the two species are distantly related and likely diverged from each other over 60 million years ago [45].

**Fig. 6:**
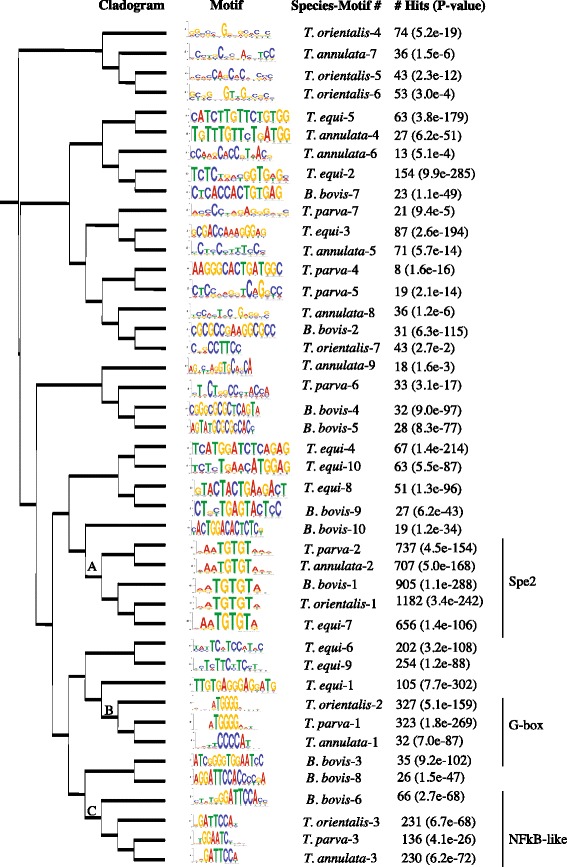
The relationships of motifs found in *T. annulata, T. equi, T. orientalis, T. parva*, and *B. bovis* intergenic regions. The tree was created with STAMP and the tree generated with iTOL (Methods). A = Spe2 motif; B = G-box motif; C = NFkB-like motif

**Fig. 7:**
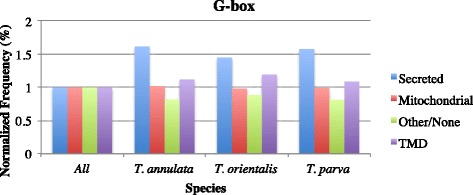
*Theileria* genes regulated by the G-box motif are enriched in genes that encoded secreted or transmembrane proteins

**Fig. 8:**
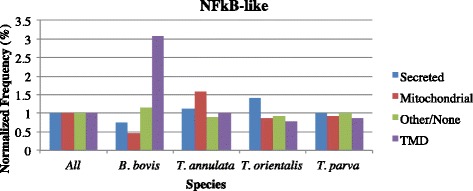
*B. bovis* genes regulated by the NFkB-like motif are enriched in genes that encoded transmembrane proteins

In order to assess the conservation of the primary upstream *cis* regulatory motifs identified in the piroplasmids, we extracted all intergenic regions 8-300 bp long from four *Theileria* and one *Babesia* species, *B. bovis*, and searched for over-represented motifs and their conservation across species. In contrast to the conservation of possibly multiple ApiAP2 binding motifs across *Plasmodium* species, among the most common motifs found in *Theileria* species, only three are shared by two or more species (Fig. 6). These are the same three most conserved and significant motifs in *T. parva*, including the G-box and the Spe2 motifs. In addition, we find that these motifs do not regulate the same gene networks (Additional file 2: Table S26), suggesting a motif turnover pattern similar to that found in other eukaryotes [46]. Out of these three motifs, we found that the G-box motif is enriched upstream of secreted or transmembrane protein-encoding genes in *T. annulata* and *T. orientalis*, as well as in *T. parva* [11], although this motif is not found among the top 10 motifs in *T. equi* (Fig. 7). Since secreted and transmembrane pathogen proteins are often immunogenic, the association with the G-box motif may have important implications for the study of interactions between *Theileria* species and their hosts. Finally, the NFkB-like motif was found upstream of transmembrane motifs in *B. bovis*, but not in *Theileria* species, which may indicate a distinct divergence in function between these piroplasmid gene networks (Fig. 8). The fact that only two motifs are common to all *Theileria* species, and that the sets of genes downstream of each motif differs between species, indicates that in fact both motifs and gene networks evolve at a significant rate in this genus, suggestive of scenario (3).

**Fig. 9:**
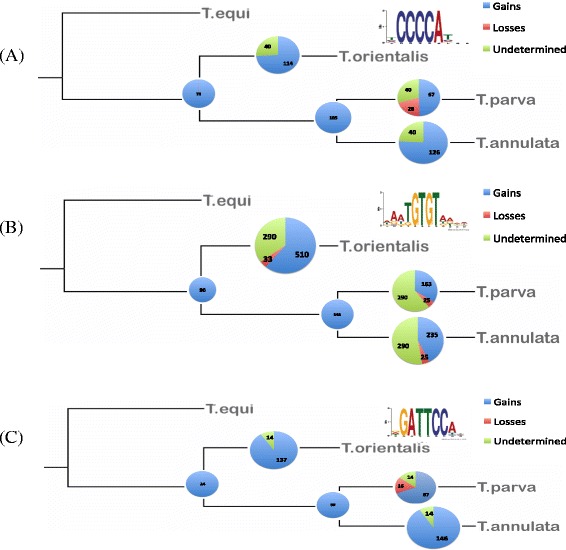
Reconstruction of turnover rate of three motifs from the present study. **a** G-box motif; **b** Spe2 motif; **c** NFkB-like motif. For each occurrence of a motif upstream of a gene in *T. parva* (Tp), *T. annulata* (Ta), *T. orientalis* (To) and *T. equi* (Te), we determined its distribution upstream of the ortologs in the other species, and infer the most parsimonious scenario for the number of acquisitions and losses of the motif in the branches leading to To, Ta and Tp. For example, a motif present upstream of the same ortholgous gene in To, Ta and Tp but absent in Te, is inferred to have arisen in the lineage leading to all the first three species, and hence be present at the node reflecting their most recent common ancestor. Motifs present in Te and one of the other species could have been lost twice, or lost once and regained, and so are deamed “undetermined”

In order to further investigate the rate of motif turnover in these piroplasms, we looked at the G-box, Spe2 and NFkB-like motifs as prototypical piroplasm upstream regulatory motifs. For each set of orthologous genes present in the four *Theileria* species for which at least one is downstream of one of these motifs, we mapped the number of times each motif was gained or lost (Fig. 9). Despite the fact that we only have available a limited number of taxa for this analysis, a few patterns are clear. First, motif gain is much more prevalent than motif loss, and that in most instances the presence of a motif upstream of a specific gene is a species-specific occurrence. Also, *T. parva* appears more prone to motif loss than *T. annulata* (defined by cases in which *T. orientalis* and *T. annulata*, but not *T. parva*, share a motif upstream of an orthologous gene). Finally, the Spe2 motif seems to be more labile than the other two, with a hundreds of instances of gains and possibly losses (Fig. 9).

Our analyses show that, overall, motifs are most conserved among the most closely related species, *T. parva* and *T. annulata*, and that their sequence conservation decreases with taxonomic distance. *T. equi* is evolutionarily quite distant from the other *Theileria* species and exhibits fundamental biological differences [18], and it is perhaps not surprising that is differs in its complement of regulatory motifs. The difference between the pattern seen among species in the genus *Plasmodium* and those in the order Piroplasma could be a reflection of either the older age of the most recent common ancestor of the Piroplasma taxa, a more conserved functional homology genome-wide within the genus *Plasmodium* than across the piroplasmids, or a combination of both. A potential caveat to this conclusion is the possibility that the annotation of some of the piroplasmid genomes contain significant errors, which prevent the identification of promoter sequences. As the quality of genome annotations improve, and more *Theileria* genomes become available, an analysis with additional granularity will facilitate a more accurate quantification of motif turnover.

## Conclusions

The highly compact nature of the *Theileria* genome seems to have resulted in complex regulation of gene transcription, such as the apparently critical role of antisense transcription of adjacent, overlapping genes. While there is an absence of canonical eukaryotic promoter elements, there do seem to be a small set of conserved promoter sequences that regulate large gene networks. While those networks differ among *Theileria* species, the G-box motif appears to be critical to the regulation of genes involved in host-pathogen interactions.

## Methods

*T. parva* RNA was isolated from a culture of an available infected bovine lymphocyte line (Tretina and Silva, in prep). No live animals were used in this research. This work required no ethical approval.

The analyses relied on the genome assembly and annotation of *T. annulata*, *B. bovis, T. equi*, *T. orientalis* available through PiroplasmaDB (piroplasmadb.org) release 23. For *T. parva*, we used the extensively updated genome annotation, described elsewhere . In short, a wide array of lines of evidence were used to improve the annotation of the most prevalent isoform for each *T. parva* gene. The sources of evidence include RNAseq read alignments and transcript assemblies, trainable and *de novo* gene prediction programs, and protein alignments and expressed sequence tags from *T. annulata* and other apicomplexans. 5′ untranslated regions (UTRs) were defined as the genomic sequence between the annotated transcription start sites (TSS) and the translation initiation codon, and 3′ UTRs were similarly defined as the genomic sequence between the translation termination codon and the annotated transcription termination site (TTS). Transcription initiation and terminations sites were defined by the presence of an uninterrupted set of reads mapped to the genome, which has a defined start and end (i.e., this set of reads does not overlap a flanking transcriptional unit encoded in the same strand). An upstream open reading frame (uORF) was defined as any open reading frame in the 5′ UTR that starts with a methionine, but that is out of frame with the primary product encoded by the locus.

Searches with the MEME program [47] used motif length constraints of 5 bp to 15 bp, searching both strands at the DNA level for a maximum of 10 motifs with an E-value cutoff of 0.05 assuming zero or one occurrence of a motif as has been done previously [11]. Motif search regions are indicated in the text. All searches with FIMO [48] shown had a p-value cutoff of 0.0001. MAST [47] searches were run with the “-hit_list” option and an e-value cutoff of 100. Meme motif position-specific scoring matrices were aligned in STAMP to identify motif similarity to newly discovered and known motifs [49]. Motifs were searched against a collection of databases including two comprehensive eukaryotic motif databases JASPAR [50] and TRANSFAC [51], FLY [52], FlyReg [53] and Agris [54], AthaMap [55], PLACE [56], Uniprobe [57], other yeast motifs [58, 59], as well as the prokaryotic motif databases RegTransBase [60] and DPInteract [61]. The Smith-Waterman local alignment method with a similarity score defined by Pearson’s correlation coefficient estimated motif similarity. The STAMP motif phylogenetic tree was generated in iTOL [62].

Orthologous clusters (OCs) were created using the predicted peptide sequences for *T. annulata*, *T. equi*, *T. orientalis, T. parva* and *B. bovis.* Jaccard-filtered OC analysis and multigene family clustering were used to construct the final ortholog set as described previously [16]. In brief, an all-versus-all BLASTP was performed, followed by orthology assignments when two proteins had an E-value ≤10-5 with ≥ 80 % similarity over ≥ 70 % of the gene and a Jaccard coefficient cutoff of 0.6.

## Availability of supporting data

The *T. parva* schizont-stage RNA-seq data is available in the NCBI Sequence Read Archive (BioSample accession SAMN03981746).

## Additional files

Additional file 1:
**Supplemental figures with information on motif composition and distribution around transcription start and termination sites, UTRs and introns.** (PDF 9.92 MB) Additional file 2:
**Supplemental tables with information on motif composition and distribution relative to genes and gene ontology.** (PDF 4.11 MB)

## Abbreviations

PAS: polyadenylation signal
SVSP: subtelomere-encoded variable secreted protein
TpHN: *T. parva* host nucleus
TSS: transcription start site
TTS: transcription termination site
uORF: untranslated open reading frame
UTR: untranslated region
