# Supplementary material for: Cis regulatory motifs and antisense transcriptional control in the apicomplexan Theileria parva
Source: BMC Genomics. 2016 Feb 20;17:128. doi: 10.1186/s12864-016-2444-5 (PMC4761415; doi:10.1186/s12864-016-2444-5)
Supplement: Additional file 1: — Supplemental figures with information on motif composition and distribution around transcription start and termination sites, UTRs and introns. (PDF 9.92 MB) [file 12864_2016_2444_MOESM1_ESM.pdf]

Additional File 1. Supplemental Figures.
Tretina, Pelle and Silva.

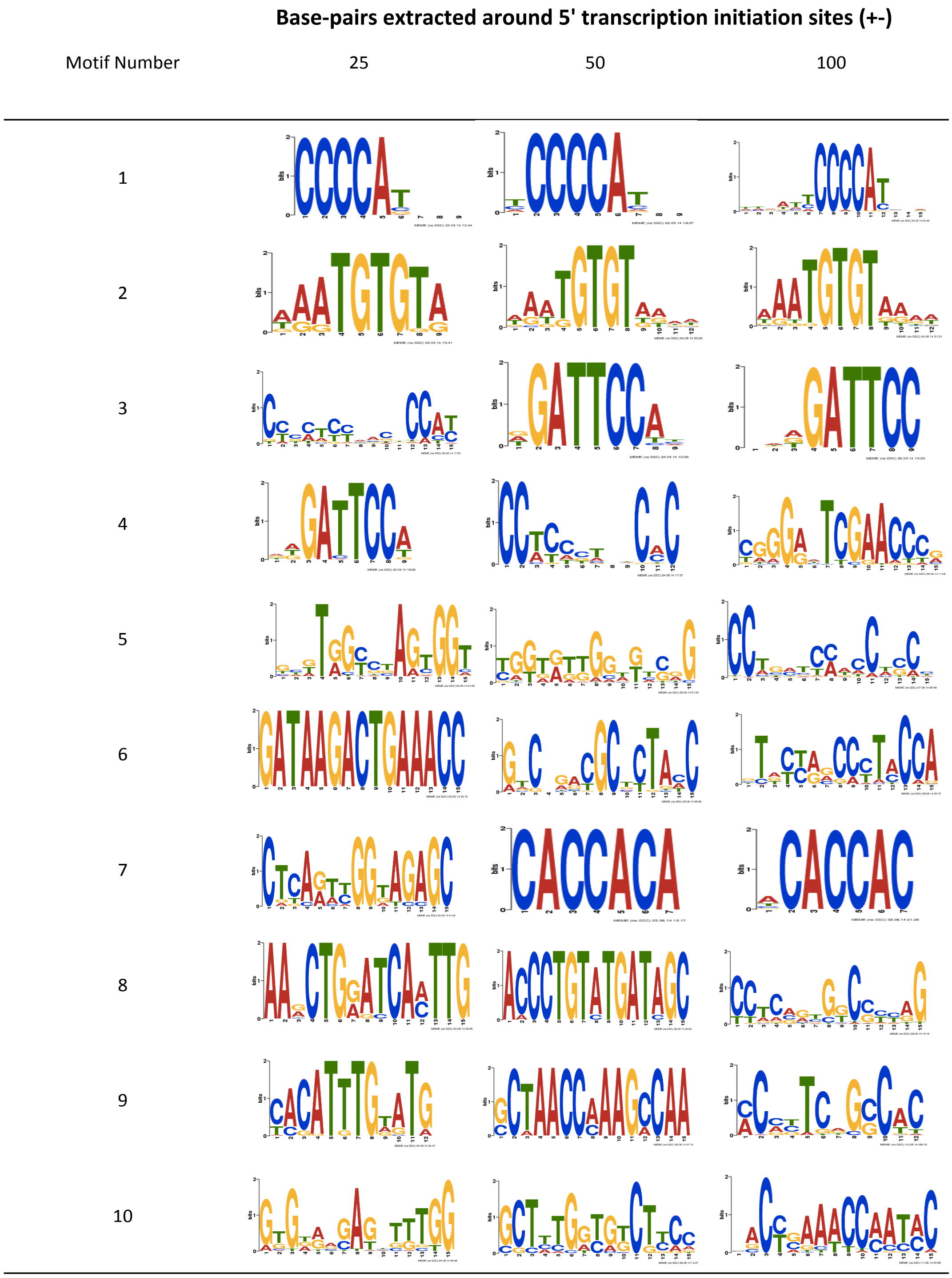

Supplemental Figure 1a. All motifs discovered around transcription initiation sites, at varying distances, and their frequency distributions.

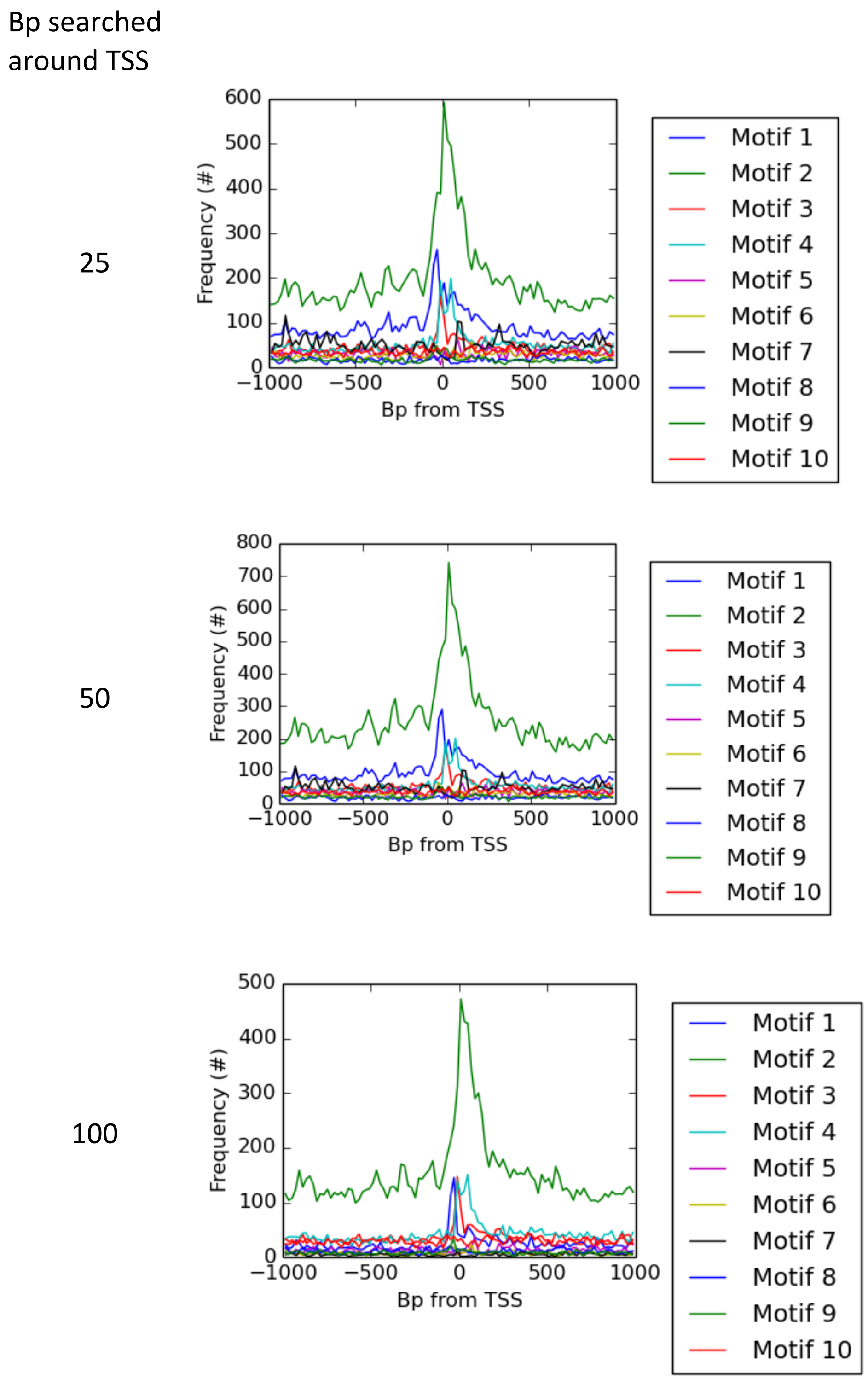

Supplemental Figure 1b. All motifs discovered around transcription start sites, at varying distances, and their frequency distributions.

	Base-pairs extracted around 3' transcription termination sites (+-)		
Motif Number	25	50	100
1	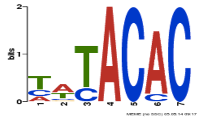	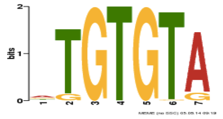	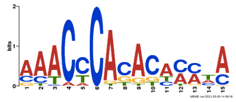
2	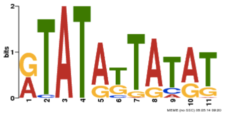	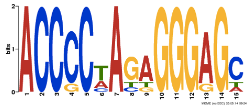	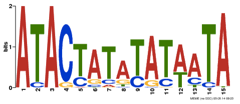
3	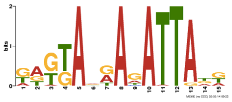	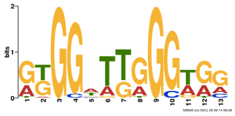	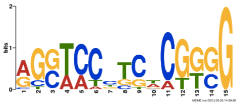
4		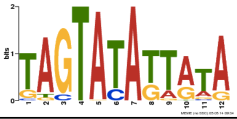	

Supplemental Figure 2a. All motifs discovered around transcription termination sites at varying distances, and their distributions.

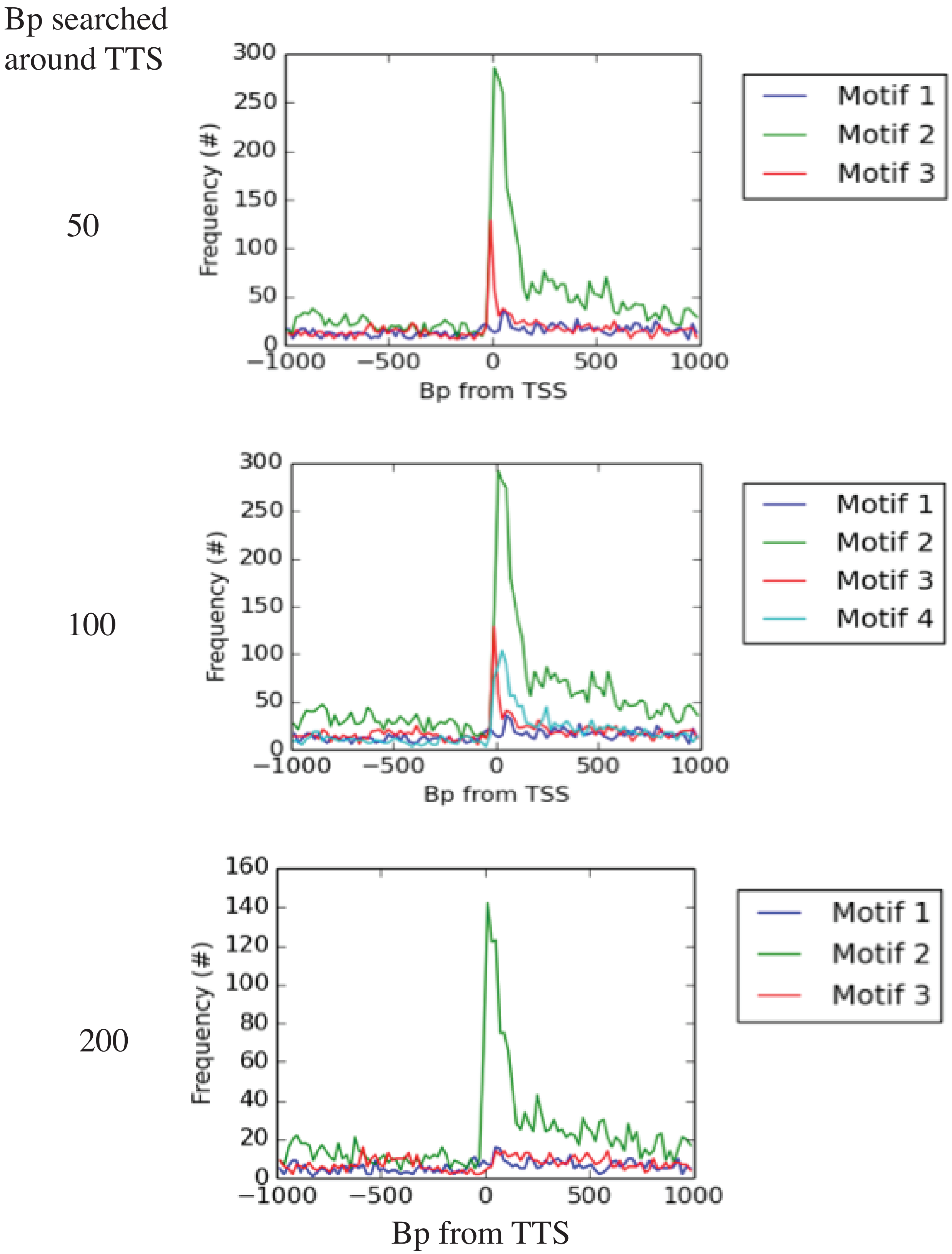

Supplemental Figure 2b. All motifs discovered around transcription termination sites at varying distances, and their distributions.

Motif

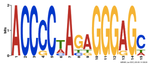

Structure Name	Image	ΔG(kcal.mole^{-1})	T_m (°C)	ΔH(kcal.mole^{-1})	ΔS(cal.K^{-1}mole^{-1})
1	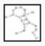	-2.99	62.5	-26.8	-79.85
2	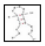	-2.64	55.4	-28.5	-86.75

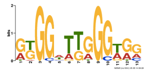

Structure Name	Image	ΔG(kcal.mole^{-1})	T_m (°C)	ΔH(kcal.mole^{-1})	ΔS(cal.K^{-1}mole^{-1})
1	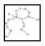	1.84	-68.8	-4	-19.58
2	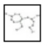	2.71	-273.1	0	-9.09

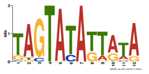

Structure Name	Image	ΔG(kcal.mole^{-1})	T_m (°C)	ΔH(kcal.mole^{-1})	ΔS(cal.K^{-1}mole^{-1})
1	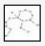	1.25	-7	-10.4	-39.08
2	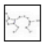	1.52	-54.3	-4.2	-19.19
3	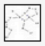	1.73	-17.4	-10.4	-40.67
4	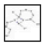	1.85	-21.6	-10	-39.76
5	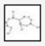	2.11	-55.5	-5.7	-26.18
6	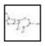	2.12	-81.9	-3.8	-19.87

Supplemental Figure 2c. Each motif from the MEME search using + 50bp sequences around the transcription termination site, and the consensus sequence submitted to Integrated DNA Technology's OligoAnalyzer 3.1 (https://www.idtdna.com/calc/analyzer) and the output displayed above.

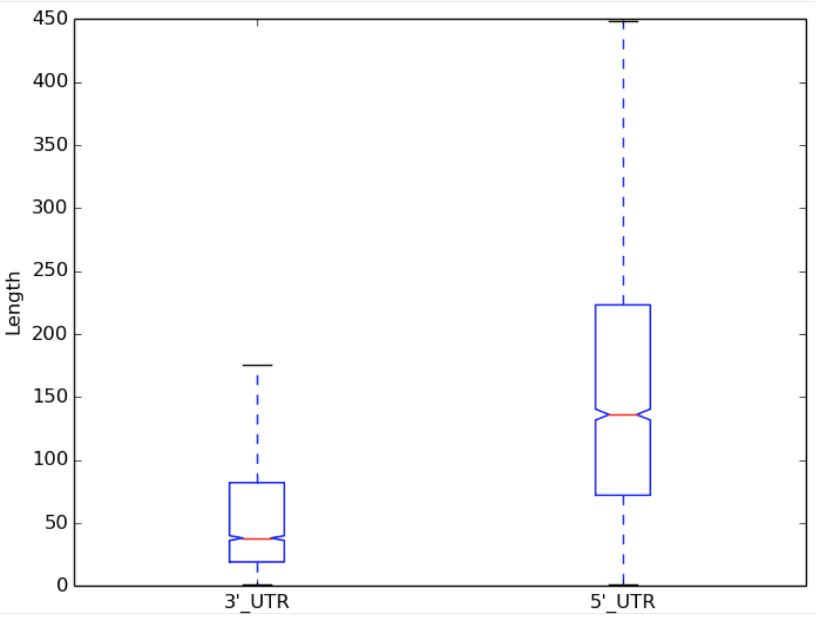

Supplemental Figure 3. Length of annotated 3´ and 5´ untranslated regions (UTRs) in *T. parva*. Box and whisker plots showing that 5´ UTRs are significantly longer than 3' UTRs. Outliers are not shown, to simplify the visual display.

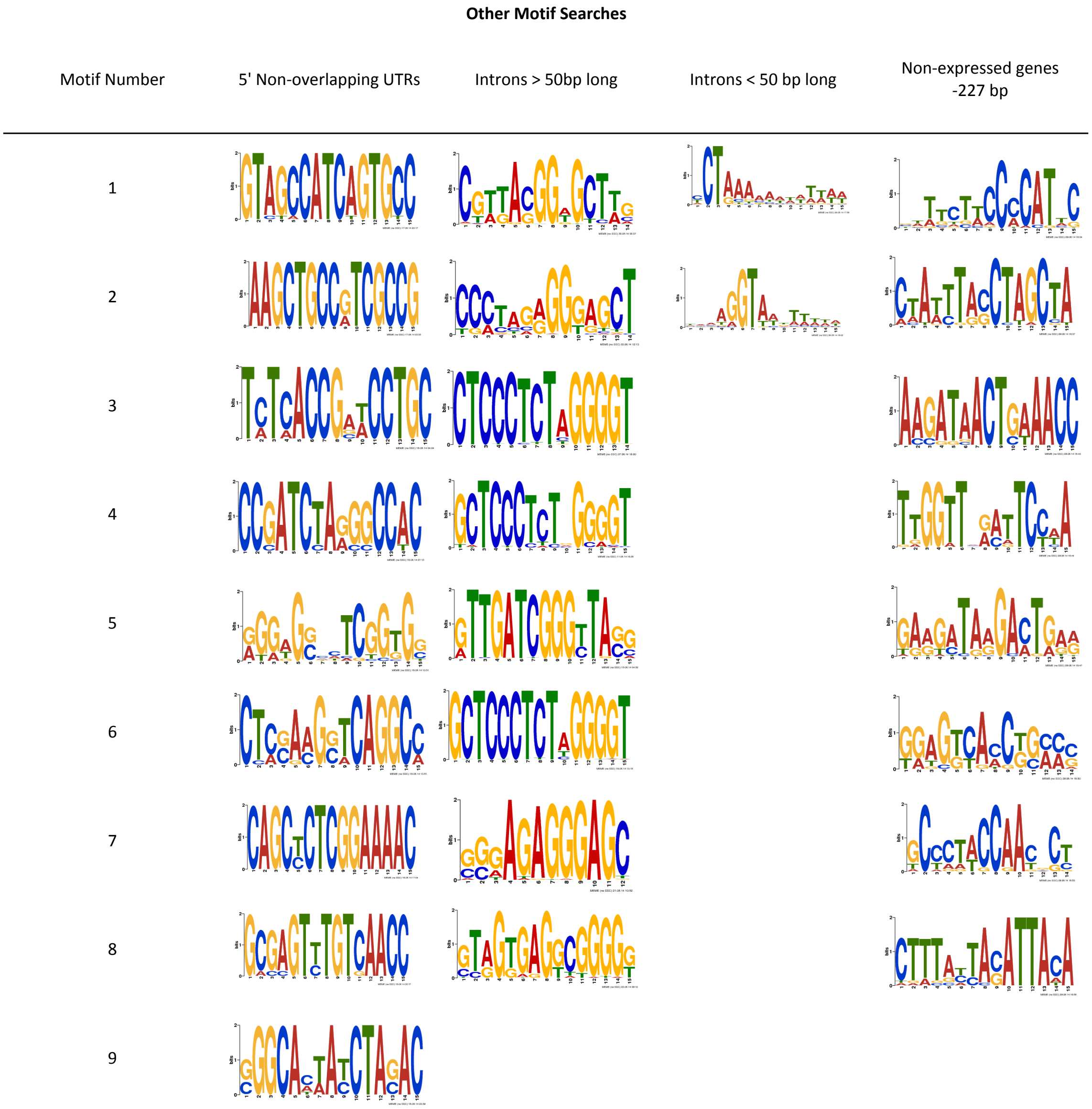

Supplemental Figure 4a. Other MEME motif searches found novel motifs. Regions that were searched include all 5' untranslated regions that did not overlap any adjacent gene, introns longer than 50bp long, introns less than 50bp long, and non-expressed genes (FPKM < 1).

Region	Consensus Motif
25bp TSS	CCCCATNNN
	AAATGTGTA
	CCCCTCCAACNCCAT
	AAGATTCCA
	GTGTGGCCTAGTGGT
	GATAAGACTGAAACC
	CTCAGTTGGTAGAGC
	AAGCTGGATCAATTG
	CACATTTGTATG
	GTGTAAGAGATTTGG
50bp TSS	TCCCCATNN
	AAATGTGTAAAA
	CGATTCCAT
	CCTCCCTNNCAC
	TGGTGTTGGTGTCGG
	GTCNGACGCTCTACCCACCACA
	ACCCTGTATGATAGC
	GCTAACCAAAGCCAA
	GCTTTGGTGTCTTCC
100bp TSS	TTCATTCCCCATCNA
	AAATGTGTAAAA
	NAAGATTCC
	CGGGAATCGAACCCG
	CCTGATCCACCTCCC
	GTACTAGCCCTACCA
	ACACCAC
	CCTCAGTGGCCCCAG
	CCCTTCAGCCAC
	NACCGAAACCAATAC

Region	Consensus Motif
25bp TTS	TATACAC
	GTATATTATAT
	GAGTANGAGATTA
50BP TTS	ATGTGTA
	ACCCCTAGAGGGAGC
	GTGGATTGGGTGG
	TAGTATATTATA
100bp TTS	AAACCCACACACCTA
	ATACTATATATAATA
	AGGTCCCTCTCGGGG
5' Non-overlapping UTRs	GTAGCCATCAGTGCC
	AAGCTGCCGTCGCCG
	TCTCACCGATCCTGC
	CCGATCTAGGGCCAC
	GGGAGGCCTCGGTGG
	CTCGAAGGTCAGGCC
	CAGCTGTCGGAAAAC
	GCGAGTTTGTCAACC
	GGGCACTATCTAGAC
Introns > 50bp long	CGTTACGGAGCTTG
	CCCTAGAGGGAGCT
	CTCCCTCTAGGGGT
	GCTCCCTCTGGGGGT
	GTTGATCGGGTTAGG
	GCTCCCTCTAGGGGT
	GGGAGAGGGAGC
	GTAGTGAGGCGGGGG
Introns < 50bp long	CCTAAAAAATATTAA
	TAAAGGTAATTTTTA
Non-expressed genes (-227bp)	GTTCTTCCCCATTC
	CTATTTACCTAGCTA
	AAGATAACTGAAACC
	TTGGTTNGATTCCAA
	GAAGATAAGACTGAA
	GGAGTCACCTGCCC
	GCCCTACCAACNCT
	CTTTATTACATTACA

Supplemental Figure 4b. This table summarizes all motifs found in the present study.
