# Supplementary material for: Cis regulatory motifs and antisense transcriptional control in the apicomplexan Theileria parva
Source: BMC Genomics. 2016 Feb 20;17:128. doi: 10.1186/s12864-016-2444-5 (PMC4761415; doi:10.1186/s12864-016-2444-5)
Supplement: Additional file 2: — Supplemental tables with information on motif composition and distribution relative to genes and gene ontology. (PDF 4.11 MB) [file 12864_2016_2444_MOESM2_ESM.pdf]

Supplemental Table 1. Statistical enrichment of motifs from Figure 3 (TSS motifs 1-3) and Figure 4 (TTS motif 4) in different kinds of intergenic regions in each *Theileria* genome. This table shows the results of two-sided Pearson's chi-square test for each comparison (red = p-value < 0.05).

Theileria parva

	Comparisons		
Motif	5'5' vs 5'3'	5'3' vs 3'3'	5'5' vs 3'3'
TSS 1	8.73E-01	4.93E-12	3.94E-10
TSS 2	7.80E-02	8.27E-06	8.57E-08
TSS 3	0.41515994	7.99E-01	7.39E-01
TTS 4	1.20E-03	5.32E-17	1.40E-21

Theileria annulata

Motif	5'5' vs 5'3'	5'3' vs 3'3'	5'5' vs 3'3'
TSS 1	3.87E-02	1.04E-17	2.27E-22
TSS 2	3.15E-18	1.45E-37	1.36E-75
TSS 3	1.00E+00	8.53E-01	1.00E+00
TTS 4	3.31E-01	3.39E-05	9.34E-03

Theileria orientalis

Motif	5'5' vs 5'3'	5'3' vs 3'3'	5'5' vs 3'3'
TSS 1	1.37E-01	8.92E-22	1.25E-25
TSS 2	9.60E-06	3.13E-19	5.98E-33
TSS 3	2.07E-01	3.56E-01	8.71E-01
TTS 4	1.61E-02	5.34E-01	1.32E-01

Theileria equi

Motif	5'5' vs 5'3'	5'3' vs 3'3'	5'5' vs 3'3'
TSS 1	3.05E-08	7.46E-22	4.60E-38
TSS 2	1.25E-21	8.13E-39	1.51E-80
TSS 3	2.20E-09	2.57E-04	2.42E-14
TTS 4	7.37E-03	2.54E-07	1.86E-11

Supplemental Table 2. Hexamers that potentially act as polyadenylation signals in T. parva.

Iterations	Top Hexamer
1	AAAAUA
2	UAAUUU
3	AAAAUU
4	UAAUAU
5	UUUAAA

Supplemental Table 3. The top 10 most frequent GO terms found in the T. parva Muguga functional annotation

**GO Term**	**Frequency**	**Name**
GO:0005524	337	ATP binding
GO:0005634	228	nucleus
GO:0005737	200	cytoplasm
GO:0016021	121	integral component of membrane
GO:0003677	110	DNA binding
GO:0046872	109	metal ion binding
GO:0003723	106	**RNA binding**
GO:0005829	103	cytosol
GO:0003735	96	structural constituent of ribosome
GO:0005730	96	nucleolus

Supplemental Table 4. Motifs found upstream of gene family 1, Subtelomere-encoded Variable Secreted Protein (SVSP) family.

	**Bp extracted around gene family 1 transcription initation sites**		
Motif Number	25	50	100
1	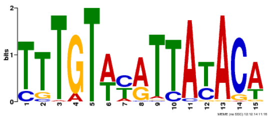	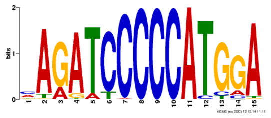	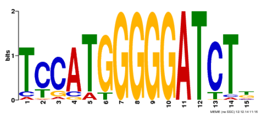
2	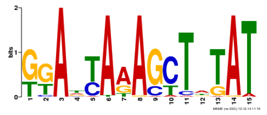	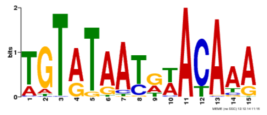	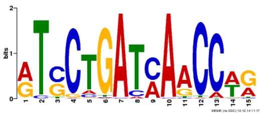
3	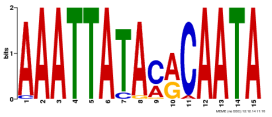	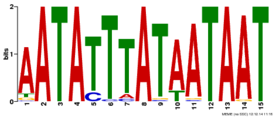	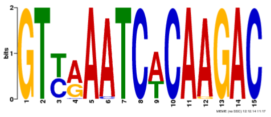
4	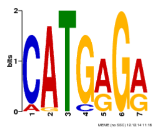	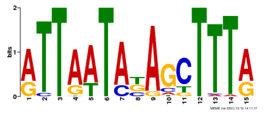	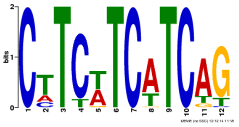
5		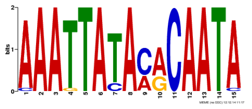	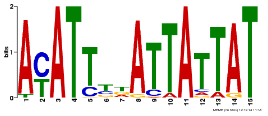
6			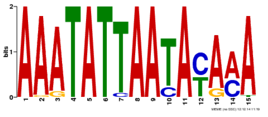
7			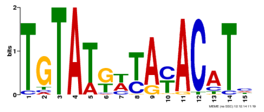
8			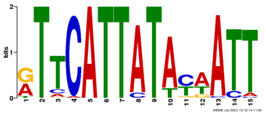
9			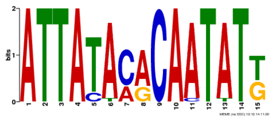
10			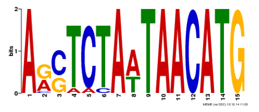

Supplemental Table 5. Motifs found upstream of gene family 2, Hypothetical DUF529 Gene family.

	**Bp extracted around gene family 2 transcription initation sites**		
Motif Number	25	50	100
1	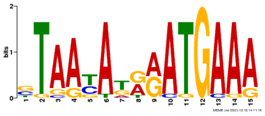	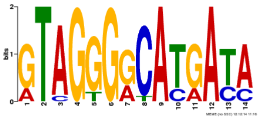	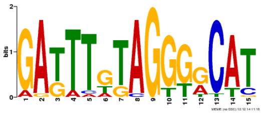
2	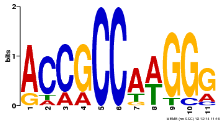	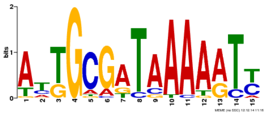	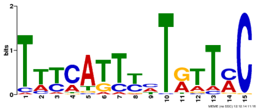
3	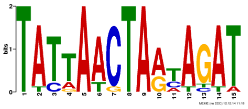	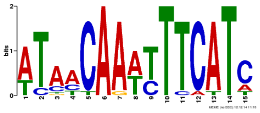	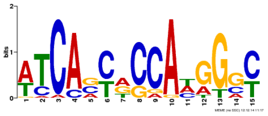
4		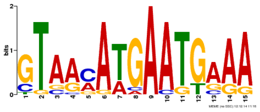	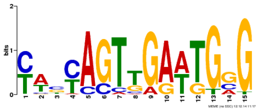
5		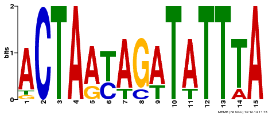	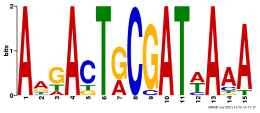
6		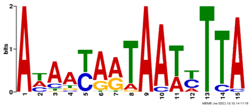	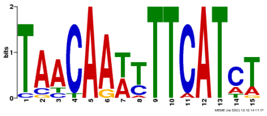
7			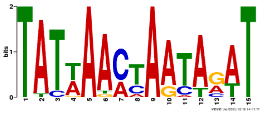

Supplemental Table 6. Motifs found upstream of gene family 3, Protein Tyrosine Kinase family.

	**Bp extracted around gene family 3 transcription initation sites**		
Motif Number	25	50	100
1	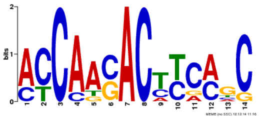	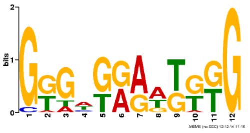	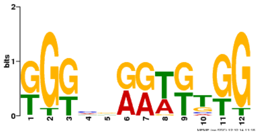

Supplemental Table 7. Motifs found upstream of gene family 4, *T. parva* Repeat (Tpr) family.

	Bp extracted around gene family 4 transcription initation sites		
Motif Number	25	50	100
1	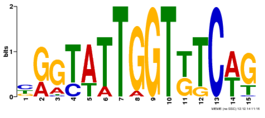	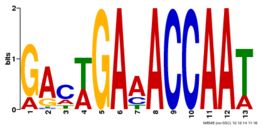	
2	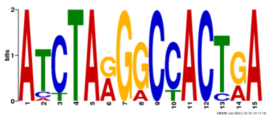	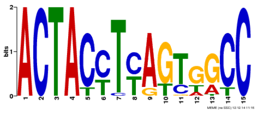	
3	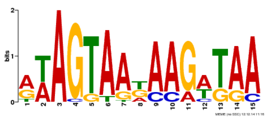	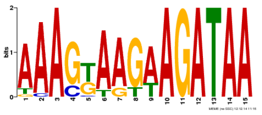	
4	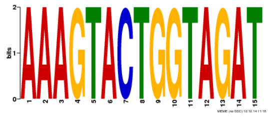	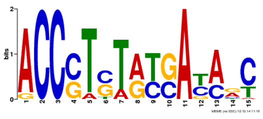	
5		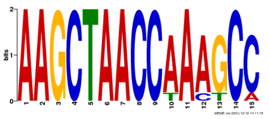	
6		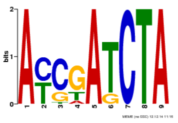	
7		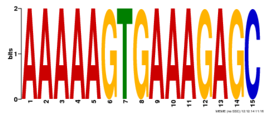	

Supplemental Table 8. Motifs found upstream of gene family 5, DEAD/DEAH Box Helicase family.

	Bp extracted around gene family 5 transcription initation sites		
Motif Number	25	50	100
1		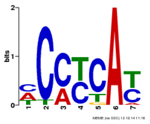	

Supplemental Table 9. Motifs found upstream of gene family 6, *T. parva* Host Nucleus (TpHN) family.

	Bp extracted around gene family 6 transcription initation sites		
Motif Number	25	50	100
1	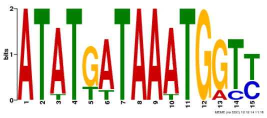	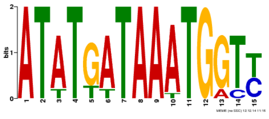	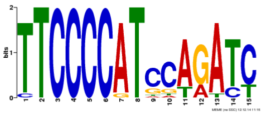
2		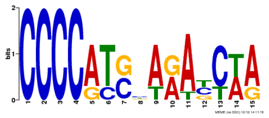	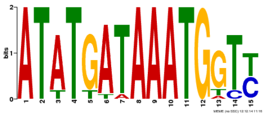
3			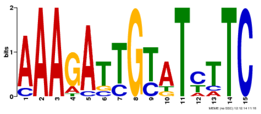
4			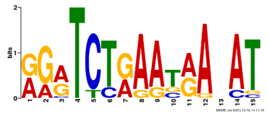
5			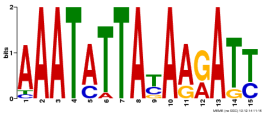
6			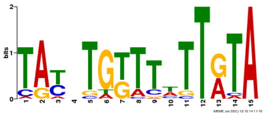

Supplemental Table 10. Motifs found upstream of gene family 7, DnaJ Chaperone family.

	Bp extracted around gene family 7 transcription initation sites		
Motif Number	25	50	100
		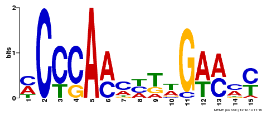	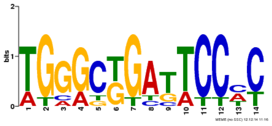

Supplemental Table 11. Motifs found upstream of gene family 8, ATPase family.

	Bp extracted around gene family 8 transcription initation sites		
Motif Number	25	50	100
1		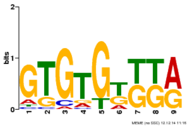	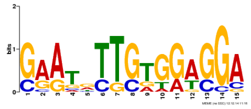

Supplemental Table 12. Motifs found upstream of gene family 9, Hypothetical Protein family.

	Bp extracted around gene family 9 transcription initation sites		
Motif Number	25	50	100
1	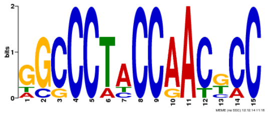	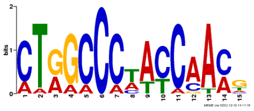	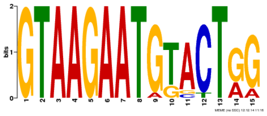
2	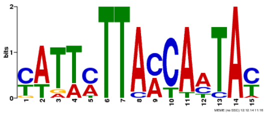	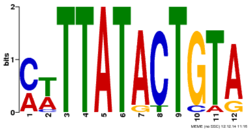	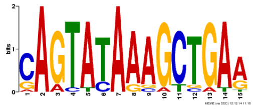
3		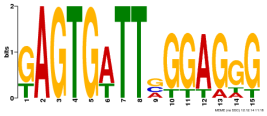	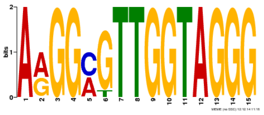
4		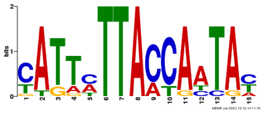	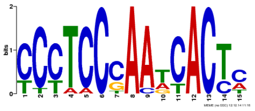
5			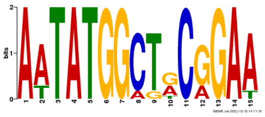
6			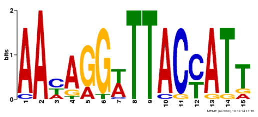
7			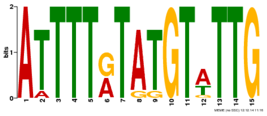
8			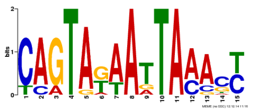

Supplemental Table 13. Motifs found upstream of gene family 10, ABC Transporter family.

	Bp extracted around gene family 10 transcription initation sites		
Motif Number	25	50	100
1	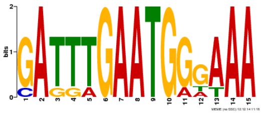	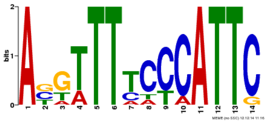	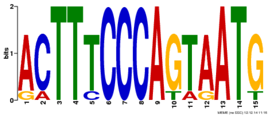
2			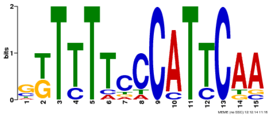

Supplemental Table 14. Motifs found upstream of gene family 11, Hypothetical Protein family.

	Bp extracted around gene family 11 transcription initation sites		
Motif Number	25	50	100
1		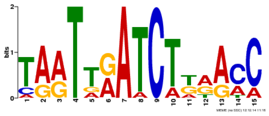	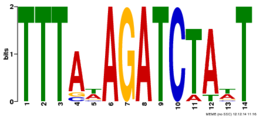
2			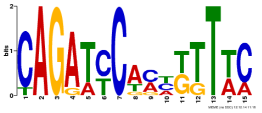

Supplemental Table 15. Motifs found upstream of gene family 12, Hypothetical Protein family.

	Bp extracted around gene family 12 transcription initation sites		
Motif Number	25	50	100
1		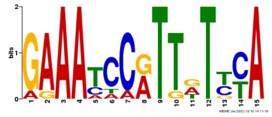	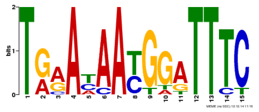

Supplemental Table 16. Motifs found upstream of gene family 13, Hypothetical DUF529 Protein family.

Motif Number	Bp extracted around gene family 13 transcription initation sites 25	50	100
1	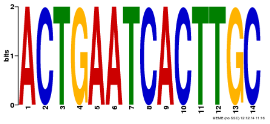	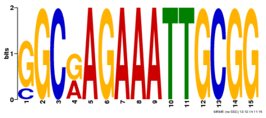	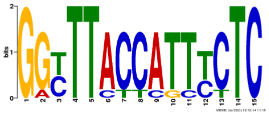
2	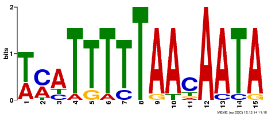	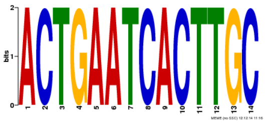	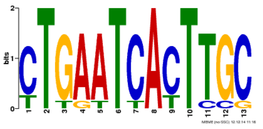
3		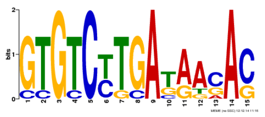	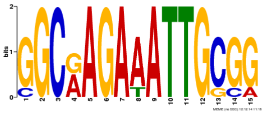
4		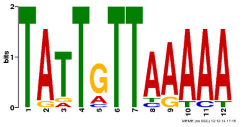	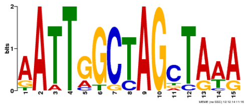
5			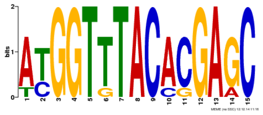
6			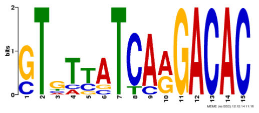
7			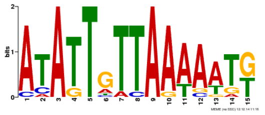

Supplemental Table 17. Motifs found upstream of gene family 14, Cyclophilin-type Peptidyl-prolyl *cis-trans* Isomerase family.

	Bp extracted around gene family 14 transcription initation sites		
Motif Number	25	50	100
1			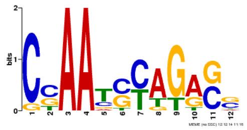

Supplemental Table 18. Motifs found upstream of gene family 15, Hypothetical Protein family.

	Bp extracted around gene family 15 transcription initation sites		
Motif Number	25	50	100
1			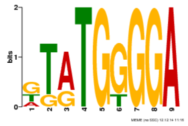

Supplemental Table 19. Motifs found upstream of gene family 20, Haloacid Dehalogenase-like Hydrolase Family.

	Bp extracted around gene family 20 transcription initation sites		
Motif Number	25	50	100
1			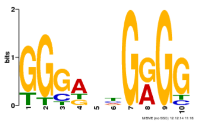

Supplemental Table 20. The distribution of motifs upsteam of the Subtelomere-encoded Variable Secreted Protein Family (SVSP) and T. parva Host Nucleus (TpHN) gene families.

	SVSP Gene Family		TpHN Gene Family
25	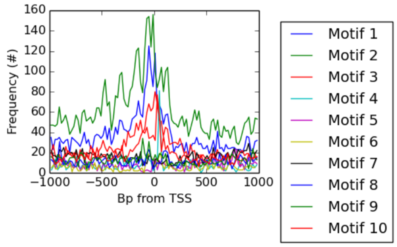	25	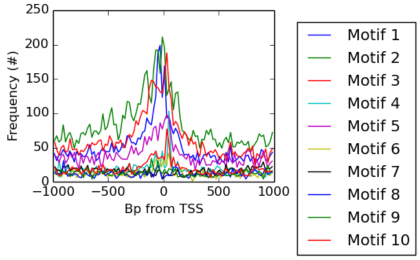
50	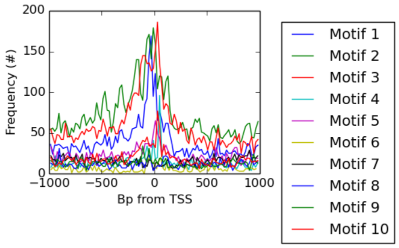	50	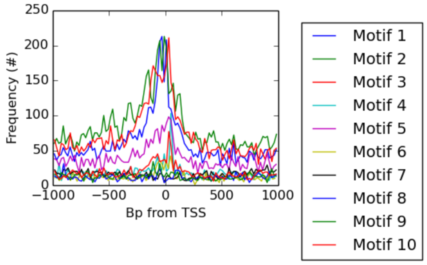
100	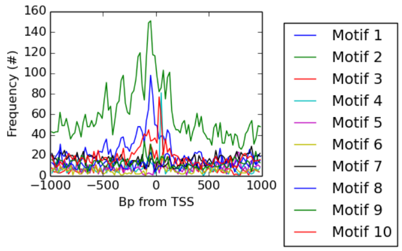	100	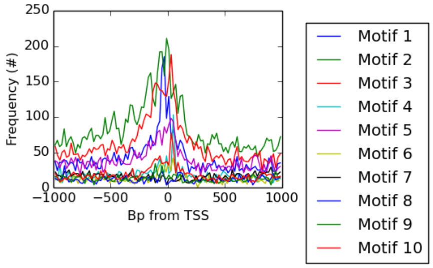

Supplemental Table 21. The genomic distribution of motifs found upstream of Subtelomere-encoded Variable Secreted Protein Family (SVSP) genes. The "search" column indicates the number of base pairs upstream and downstream of transcription initiation sites that were used for the motif search.

SVSP Family genes

Search	Motif (#)	Motif	Species Distribution
25	1	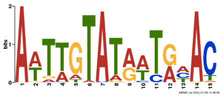	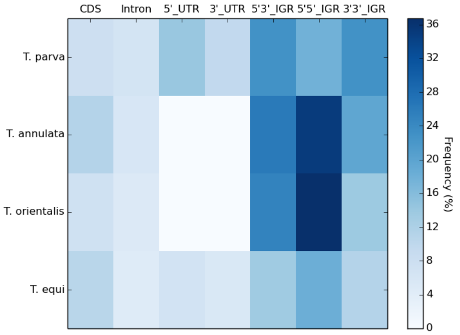
	2	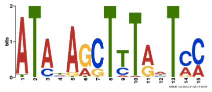	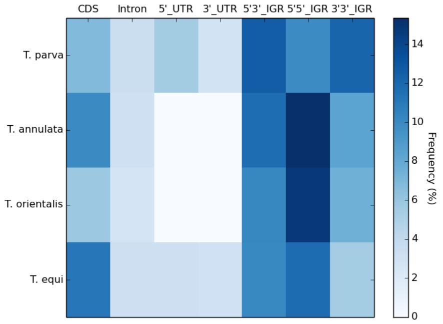
	3	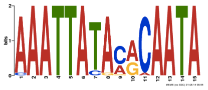	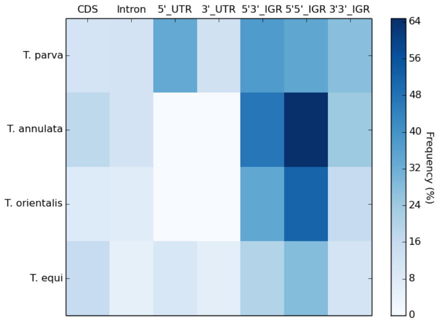
	4	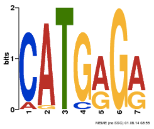	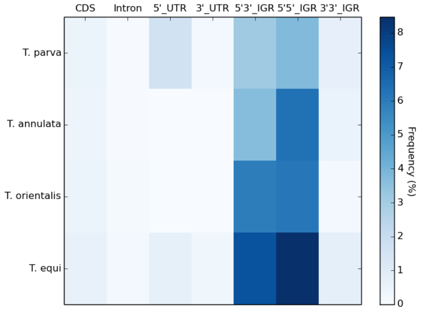

Supplemental Table 22. The genomic distribution of motifs found upstream of Subtelomere-encoded Variable Secreted Protein Family (SVSP) genes. The “search” column indicates the number of base pairs upstream and downstream of transcription initiation sites that were used for the motif search.

Search	Motif (#)	Motif	Species Distribution
50	1	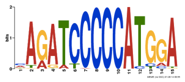	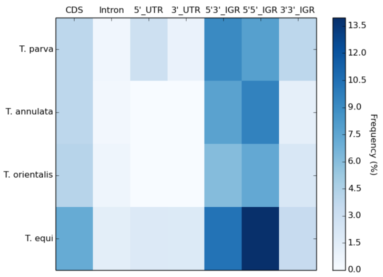
	2	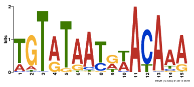	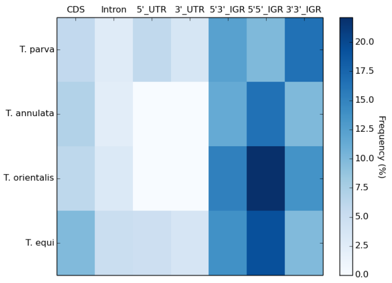
	3	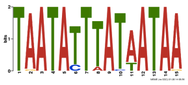	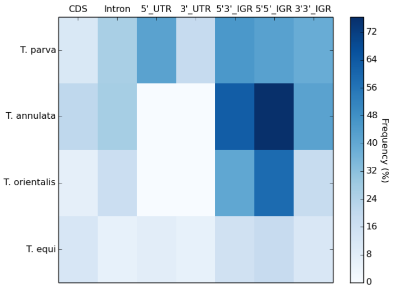
	4	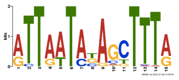	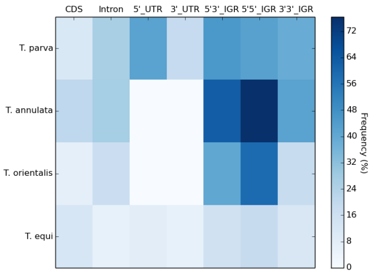
	5	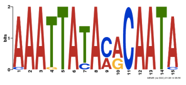	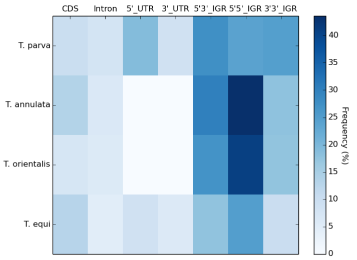

Supplemental Table 22a. The genomic distribution of motifs found upstream of Subtelomere-encoded Variable Secreted Protein Family (SVSP) genes. The “search” column indicates the number of base pairs upstream and downstream of transcription initiation sites that were used for the motif search.

SVSP Family genes

Search	Motif (#)	Motif	Species Distribution
100	1	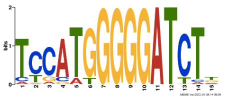	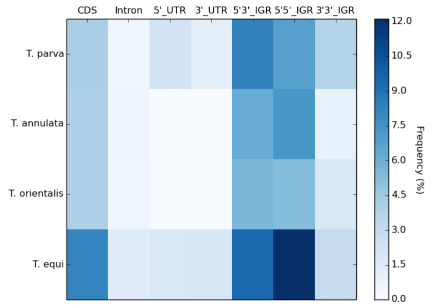
	2	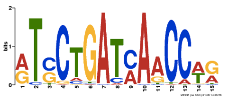	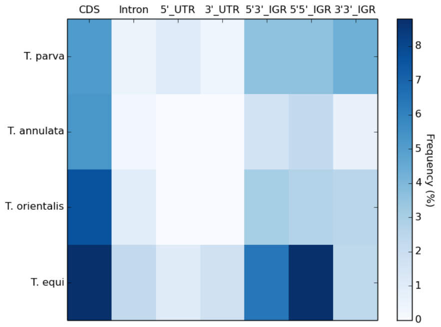
	3	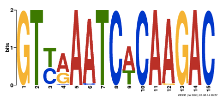	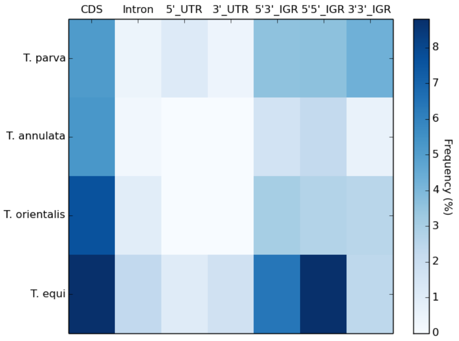
	4	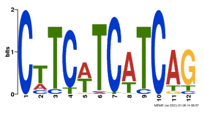	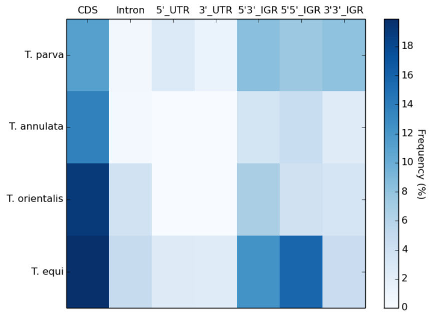

Supplemental Table 22b. The genomic distribution of motifs found upstream of Subtelomere-encoded Variable Secreted Protein Family (SVSP) genes. The “search” column indicates the number of base pairs upstream and downstream of transcription initiation sites that were used for the motif search.

SVSP Family genes

Search	Motif (#)	Motif	Species Distribution
100	5	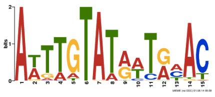	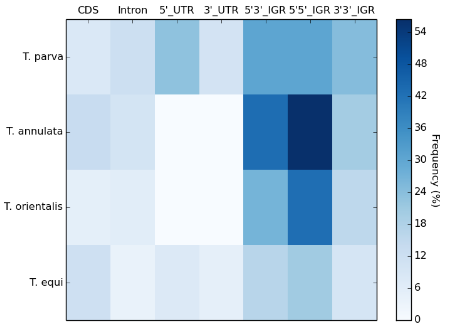
	6	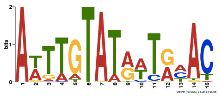	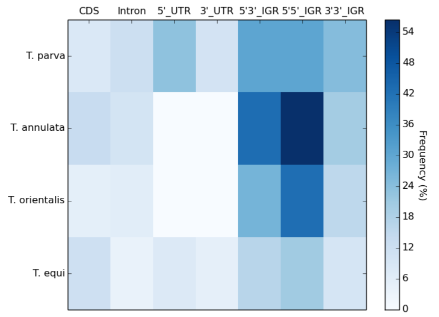
	7	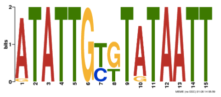	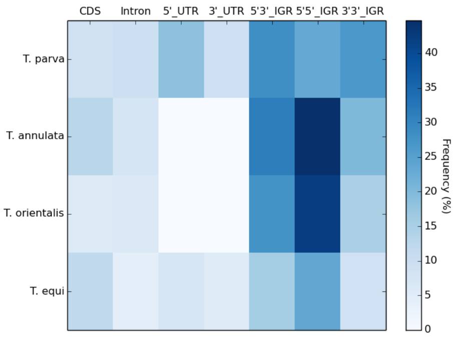
	8	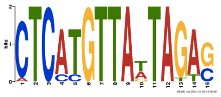	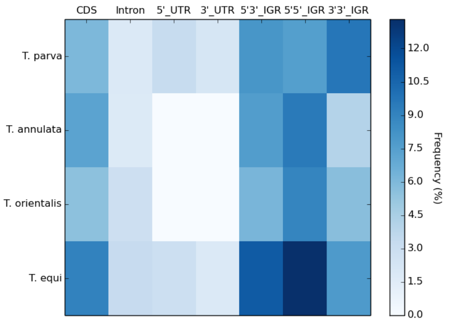

Supplemental Table 22c. The genomic distribution of motifs found upstream of Subtelomere-encoded Variable Secreted Protein Family (SVSP) genes. The “search” column indicates the number of base pairs upstream and downstream of transcription initiation sites that were used for the motif search.

SVSP Family genes

Search	Motif (#)	Motif	Species Distribution
100	9	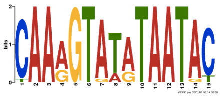	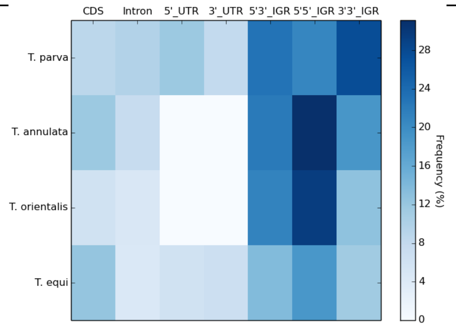
	10	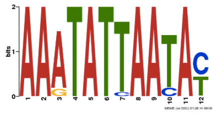	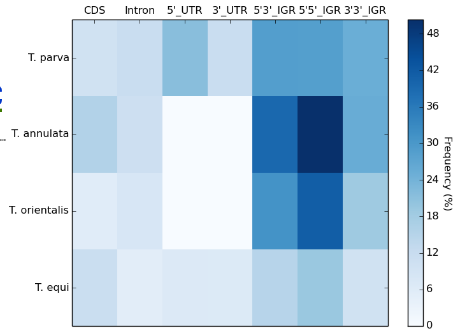

Supplemental Table 23. The genomic distribution of motifs found upstream of T. parva Host Nucleus (TpHN) genes. The "search" column indicates the number of base pairs upstream and downstream of transcription initiation sites that were used for the motif search.

TpHN Family genes

**Search**	**Motif (#)**	**Motif**	**Species Distribution**
25	1	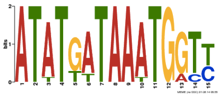	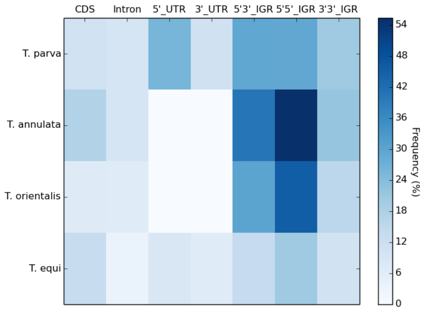

Supplemental Table 24. The genomic distribution of motifs found upstream of T. parva Host Nucleus (TpHN) genes. The “search” column indicates the number of base pairs upstream and downstream of transcription initiation sites that were used for the motif search.

TpHN Family genes

Search	Motif (#)	Motif	Species Distribution
50	1	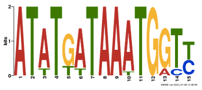	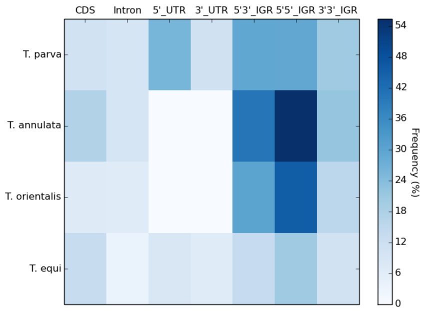
	2	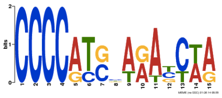	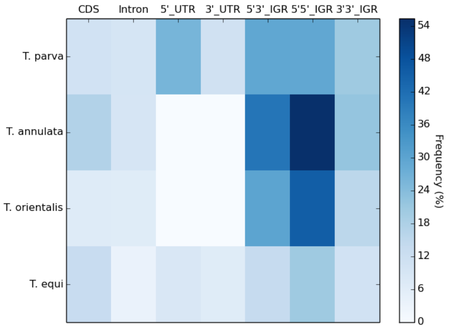
	3	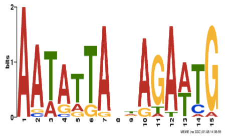	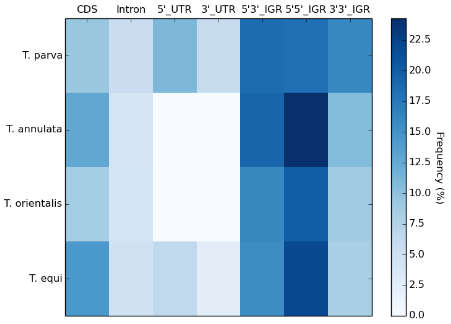

Supplemental Table 25a. The genomic distribution of motifs found upstream of T. parva Host Nucleus (TpHN) genes. The “search” column indicates the number of base pairs upstream and downstream of transcription initiation sites that were used for the motif search.

TpHN Family genes

**Search**	**Motif (#)**	**Motif**	**Species Distribution**
100	1	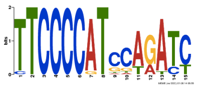	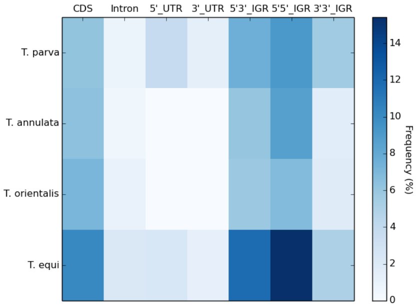
	2	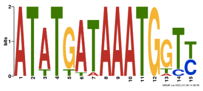	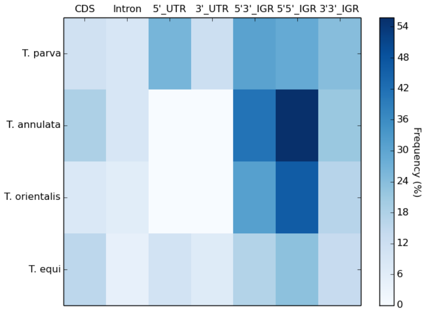
	3	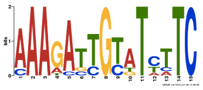	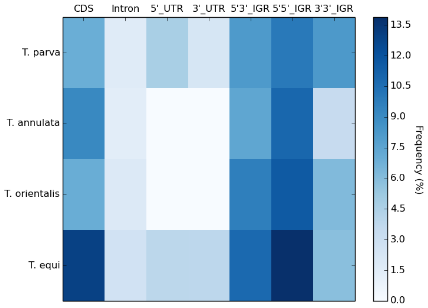
	4	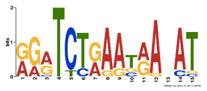	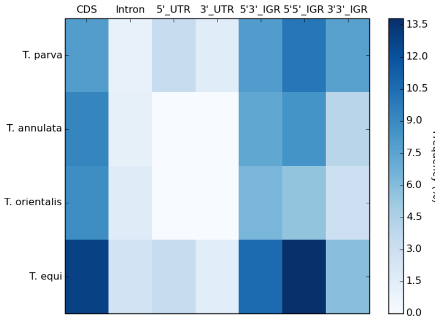

.

Supplemental Table 25b. The genomic distribution of motifs found upstream of T. parva Host Nucleus (TpHN) genes. The “search” column indicates the number of base pairs upstream and downstream of transcription initiation sites that were used for the motif search.

TpHN Family genes

**Search**	**Motif (#)**	**Motif**	**Species Distribution**
100	5	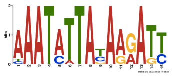	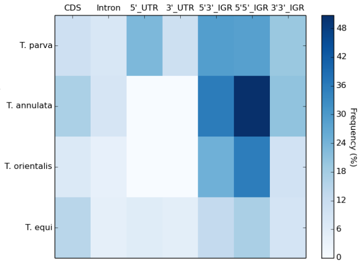
	6	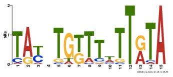	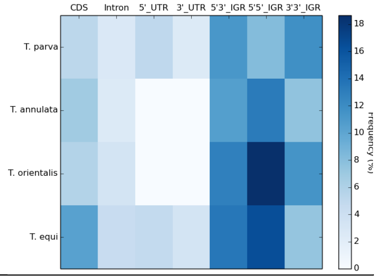

Supplemental Table 26. The conservation of gene networks downstream of the three highest-conserved motifs. Note that each table is not symmetrical across the diagonal because each species has different numbers of motifs, as well as number of orthologs for some genes.

Spe2 Percent with high-confidence orthologs and upstream motif

Species	*Bb*	*Te*	*Ta*	*To*	*Tp*	Total
Tp	17.5	13.3	37.31	30.39	100	737
To	17.85	12.01	14.89	100	18.95	1182
Ta	22.07	11.6	100	24.89	38.9	707
Te	17.38	100	12.5	21.65	14.94	656
Bb	100	12.6	17.24	23.31	14.25	905

G-box Percent with high-confidence orthologs and upstream motif

Species	*Ta*	*To*	*Tp*	Total
Tp	47.06	26.93	100	323
To	22.94	100	26.61	327
Ta	100	20.95	42.46	358

NFkB-like Percent with high-confidence orthologs and upstream motif

Species	*Bb*	*Ta*	*To*	*Tp*	Total
Tp	2.21	34.56	16.18	100	136
To	2.16	10.39	100	9.53	231
Ta	1.74	100	10.43	20.43	230
Bb	100	6.06	7.58	4.55	66
